# Supplementary material for: Causal Relationship between Aging and Anorexia Nervosa: A White-Matter-Microstructure-Mediated Mendelian Randomization Analysis
Source: Biomedicines. 2024 Aug 16;12(8):1874. doi: 10.3390/biomedicines12081874 (PMC11351342; doi:10.3390/biomedicines12081874)
Supplement: Supplementary file 1 [file biomedicines-12-01874-s001.zip › Supplementary Figure/Figure S4/Singe SNP plot of MR analysis for TL to ubm-b-1903.pdf]

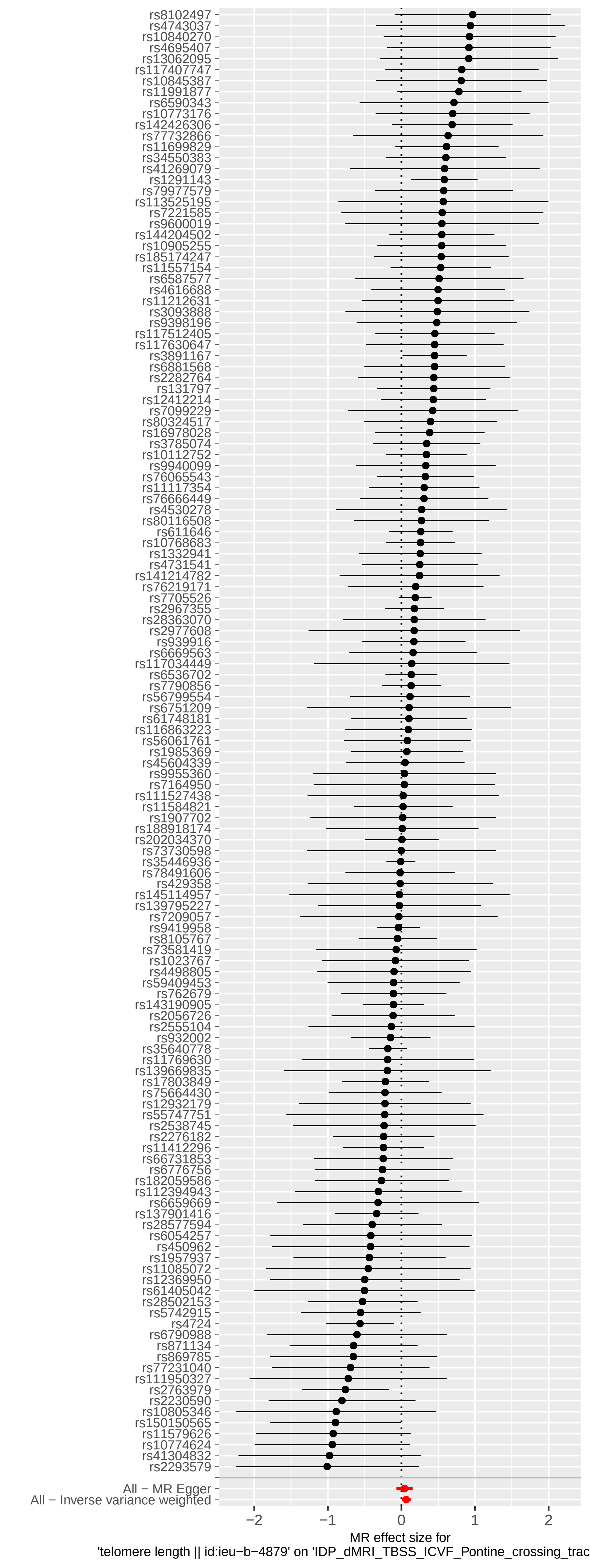
rs8102497
rs4743037
rs10840270
rs4695407
rs13062095
rs117407747
rs10845387
rs11991877
rs6590343
rs10773176
rs142426306
rs77732866
rs11699829
rs34550383
rs41269079
rs1291143
rs79977579
rs113525195
rs7221585
rs9600019
rs144204502
rs10905255
rs185174247
rs11557154
rs6587577
rs4616688
rs11212631
rs3093888
rs9398196
rs117512405
rs117630647
rs3891167
rs6881568
rs2282764
rs131797
rs12412214
rs7099229
rs80324517
rs16978028
rs3785074
rs10112752
rs9940099
rs76065543
rs11117354
rs76666449
rs4530278
rs80116508
rs611646
rs10768683
rs1332941
rs4731541
rs141214782
rs76219171
rs7705526
rs2967355
rs28363070
rs2977608
rs939916
rs6669563
rs117034449
rs6536702
rs7790856
rs56799554
rs6751209
rs61748181
rs116863223
rs56061761
rs1985369
rs45604339
rs9955360
rs7164950
rs111527438
rs11584821
rs1907702
rs188918174
rs202034370
rs73730598
rs35446936
rs78491606
rs429358
rs145114957
rs139795227
rs7209057
rs9419958
rs8105767
rs73581419
rs1023767
rs4498805
rs59409453
rs762679
rs143190905
rs2056726
rs2555104
rs932002
rs35640778
rs11769630
rs139669835
rs17803849
rs75664430
rs12932179
rs55747751
rs2538745
rs2276182
rs11412296
rs66731853
rs6776756
rs182059586
rs112394943
rs6659669
rs137901416
rs28577594
rs6054257
rs450962
rs1957937
rs11085072
rs12369950
rs61405042
rs28502153
rs5742915
rs4724
rs6790988
rs871134
rs869785
rs77231040
rs111950327
rs2763979
rs2230590
rs10805346
rs150150565
rs11579626
rs10774624
rs41304832
rs2293579
All − MR Egger
All − Inverse variance weighted
−2
−1
0
1
2
MR effect size for
'telomere length || id:ieu−b−4879' on 'IDP_dMRI_TBSS_ICVF_Pontine_crossing_trac
